# Cardiopulmonary Exercise Testing in Children With Long COVID: A Case-controlled Study

**DOI:** 10.1097/INF.0000000000004371

**Published:** 2024-05-07

**Authors:** Fabiana Baldi, Cristina De Rose, Francesco Mariani, Rosa Morello, Francesca Raffaelli, Piero Valentini, Danilo Buonsenso

**Affiliations:** * From the Pulmonary Medicine Unit, Department of Medical and Surgical Sciences; † Department of Woman and Child Health and Public Health; ‡ Dipartimento di Scienze di Laboratorio e Infettivologiche, Fondazione Policlinico Universitario A. Gemelli IRCCS; § Centro di Salute Globale, Università Cattolica del Sacro Cuore, Rome, Italy.

**Keywords:** long COVID, children, post-COVID condition, cardiopulmonary exercise testing

## Abstract

**Background:**

Cardiopulmonary exercise testing (CPET) is a noninvasive and nonexpensive diagnostic tool, that provides a comprehensive evaluation of the pulmonary, cardiovascular, and skeletal muscle systems’ integrated reactions to exercise. CPET has been extensively used in adults with Long COVID (LC), while the evidence about its role in children with this condition is scarce.

**Methods:**

Prospective, case-controlled observational study. Children with LC and a control group of healthy children underwent CPET. CPET findings were compared within the 2 groups, and within the LC groups according to main clusters of persisting symptoms.

**Results:**

Sixty-one children with LC and 29 healthy controls were included. Overall, 90.2% of LC patients (55 of 61) had a pathologic test vs 10.3% (3/29) of the healthy control. Children with LC presented a statistically significant higher probability of having abnormal values of peak VO2 (*P* = 0.001), AT% pred (*P* <0.001), VO2/HR % (*P* = 0.03), VO2 work slope (*P* = 0.002), VE/VCO2 slope (*P* = 0.01). The mean VO2 peak was 30.17 (±6.85) in LC and 34.37 (±6.55) in healthy patients (*P* = 0.007).

**Conclusions:**

Compared with healthy controls, children with LC have objective impaired functional capacity (expressed by a low VO2 peak), signs of deconditioning and cardiogenic inefficiency when assessed with CPET. As such, CPET should be routinely used in clinical practice to objectify and phenotype the functional limitations of children with LC, and to follow-up them.

SARS-CoV-2 in children is associated with a mild acute disease with full recovery in most cases.^1^ However, postacute complications have been documented in infected children throughout the pandemic. The multisystem inflammatory syndrome is a severe, hyperinflammatory disease that develops 2 to 8 weeks after initial infection,^1^ but has become exceedingly rare since the postomicron era.^2^ Long COVID (LC), or post-COVID condition, is a more subtle outcome of acute COVID-19, characterized by the persistence of one or more unexplained symptoms for 8–12 weeks after the initial infection that negatively affects daily life.^3^ The most prevalent symptoms among the many are fatigue, postexertional malaise, palpitations, muscle and joint pains, gastrointestinal disturbances and cognitive dysfunction, that negatively impact daily activities. LC has been extensively studied in adults, with multiple studies showing that adults with this condition have specific immune-metabolic biosignatures^4^ or abnormalities in several imaging or functional tests, like cardiopulmonary exercise testing (CPET).^5^

In children, despite LC has been documented by several studies from all over the world, diagnostic studies are scares and there are still clinicians that doubt about the organic or psychological nature of the condition.^6,7^ Small case series or case reports have documented abnormalities in brain metabolism,^8,9^ lung perfusion, 24 hours electrocardiogram (ECG) and CPET,^10–13^ however large case-controlled studies are still lacking. The lack of such studies limits our understanding of the disease, but also diagnostic strategies and, consequently, access to care of patients.

CPET is a well-known, noninvasive and nonexpensive diagnostic tool, that provides a comprehensive evaluation of the pulmonary, cardiovascular and skeletal muscle systems’ integrated reactions to exercise, in contrast to other diagnostic tests that evaluate one organ system. Therefore, CPET provides useful information about the pathophysiology of cardiovascular and ventilatory systems in patients with functional limitations.^14^

Given that fatigue and cardiorespiratory symptoms are frequently reported in children with LC, we hypothesized that this test would be an objective diagnostic to differentiate LC from healthy children and to characterize subphenotypes of pediatric LC.

## MATERIALS AND METHODS

### Aims

The main objective of this study was to analyze the findings of CPET in children with LC compared with healthy controls.

In particular, we attempted to:

1. Evaluate if children with LC have objective abnormalities detectable with CPET compared with health controls.
2. Understand if CPET can provide insights into the pathophysiological mechanisms underlying exercise intolerance in the wide spectrum of symptoms reported by patients with LC.
3. Provide subphenotypes of LC based on symptoms that persisted months after acute infection, correlating them with CPET results.

### Study Design

This is a prospective, single-center, case-control observational study conducted at the Policlinico Agostino Gemelli in the period between May 2021 and September 2023 in which pediatric patients (age <18 years) were enrolled and underwent CPET in presence of persistent symptoms compatible with the definition of LC according to the World Health Organization definition, requiring symptoms that last at least 2 months and cannot be explained by an alternative diagnosis, and symptoms have a negative impact on daily life.^3^ The diagnosis was made after careful in-presence clinical assessment by a clinician expert in pediatric LC as recently described.^15^ In eligible children blood tests (including complete blood cell count, liver and kidney function, glucose, tests of thyroid function, celiac disease and autoimmunity) were performed to exclude other causes of symptoms, as part of our previously published local pathways for children with LC.^15,16^ For the LC cohort, only patients who had received a diagnosis of LC within 6 months since the initial SARS-CoV-2 infection (and had symptoms for at least 3 months, according to the World Health Organization definition) were included. Patients were excluded in case of age >17 years, history of congenital cardiac defects or previous history of any other cardiac or respiratory tract disease, or if an alternative explanation for persisting symptoms was found during first-level blood tests.

LC patients were compared with a control population of 29 pediatric healthy, asymptomatic, age- and sex-matched subjects who also performed CPET during the same study period.

Demographic characteristics, including age and sex, ethnicity, weight (kg), height (cm) and BMI (kg/m^2^), were recorded. In all children, a detailed medical history, concerning both the acute phase of SARS-CoV-2 infection and the post-COVID-19 persistent manifestations, (fatigue, dyspnea, chest pain, palpitations, syncope, respiratory symptoms, headache, muscle pain, nausea or other gastrointestinal symptoms) was obtained.

### Evaluation of Functional Capacity With Cardiopulmonary Exercise Testing

CPET was conducted with an electromagnetic brake cycle ergometer. The CPETs were all performed by an ERGOSTIK TM Geratherm (@Medgraphics) cardiorespiratory device equipped with “Blue Cherry software,” regularly calibrated for gases (O_2_ and CO_2_) and volumes before each test, with breath-by-breath data acquisition, using a suitable face mask size, with continuous 12-lead ECG monitoring, finger pulse oximetry, and manual blood pressure detection at baseline and every 2 minutes during cycling.

Before starting each test, informed consent was obtained, and the SARS-CoV-2 swab was preliminarily performed as required by current hospital rules.

After acquiring the relevant clinical information, anthropometric data (age, weight and height) were entered to calculate the CPET-related parameters and the patient was explained how to carry out the test. All CPET performed for the study were conducted with a RAMP protocol with incremental load in which after the “warm-up phase,” the work rate is increased each minute or continuously for 10 ± 2 min.

According to current recommendations,^17^ the CPET procedure was divided into 4 parts:

1. Resting phase (2 min): there is an adaptation of respiration to the mask including measurements of ECG, peripheral oxygen saturation (SpO2) and blood pressure.
2. Warm-up phase (2 min): at a verbal sign the patient begins unloaded pedaling keeping a cycling speed of 70–80 rpm.
3. Incremental exercise phase (10 ± 2 min): during this phase the patient is encouraged by the nurse and physician to maintain a cadence of 65–70 rpm and maximize his performance as possible he/she can.
4. Recovery phase (2 min): unloaded pedaling.

The exams were interrupted if the patients had evidences of distress, if there was a fall in systolic blood pressure greater than 10 mm Hg, if a significant arrhythmia developed, or if the patients had ST segment depression of 3 mm or greater on ECG monitoring. The test was also stopped if the patient was unable to maintain cycling frequency above 40 rpm for exhaustion effort and muscle pains of the legs.

The following CPET measurements were studied:

1. (Peak VO2) Peak oxygen uptake expressed in absolute value (mL/kg/min) and percentage of predict value (%).
2. Respiratory exchange ratio (RER): RER is an indicator of the quality of effort. A RER >1.1 is considered maximal.
3. VCO2 [carbon dioxide production (CO_2_) production] and gas ventilatory equivalents (VE/VCO2 and VE/VO2).
4. VE/VCO2 slope: slope of the minute ventilation (VE) to CO_2_ production ratio.

Elevated value (above 30) reflects ventilatory inefficiency or ventilation/perfusion mismatch

1. Anaerobic threshold (AT) calculated by 3 methods: V slope method and ventilatory equivalent for oxygen uptake e carbon dioxide (VE/VO2 and VE/VCO2) and End-tidal pressure (PET) O_2_ and (PET) CO_2_ methods and expressed as % of predicted of VO2 (AT) % max.
2. Breathing respiratory reserve (BR) expressed as % predicted of the difference in liters and between maximum voluntary ventilation and VE peak.
3. Arterial blood oxygen saturation (SpO2) measured at basal and at peak of exercise.
4. VO2/Work rate expressed in mL/min/watt (The slope of VO2/WR reflects the metabolic conversion of chemical potential energy to mechanical work and the mechanical ability of the musculoskeletal system).
5. Oxygen pulse (VO2/HR: uptake of oxygen to heart rate ratio) expressed in % of predicted.
6. HR (heart rate) at the peak of exercise expressed of % of predicted.
7. End-tidal pressure for carbon dioxide (PET CO_2_) and ventilation minute for carbon dioxide production (VE/VCO2) calculated at the AT to estimate the likelihood of pulmonary vasculopathy involvement.^18^

### Statistical Analysis

Categorical variables were reported as count and percentage. Continuous variables were expressed as mean with standard deviation or as median with interquartile range (IQR 25%–75%). Statistical association between categorical variables was obtained by *χ*^2^ tests or Fisher exact tests while Mann–Whitney *U* test was used to compare continuous variables. *P* < 0.05 was considered statistically significant. As no other pediatric studies ever evaluated the role of CPET in LC, this study is settled as a pilot study and not sample size could be calculated.

### Ethical Approval

The study was approved by the local ethics committee (Gemelli University Hospital, Ethic approval ID4518, Prot0040139/21) and informed consent was provided. Written and informed consent was obtained from parents/caregivers and from children older than 5 years of age, according to local guidance of the ethics committees.

## RESULTS

### Study Population

In the period between May 2021 and September 2023, 90 patients in the pediatric age group were enrolled; the age of the patients was between 12 and 15 years (average age 13 years old). Of the total population studied (N = 90), 61 were diagnosed with LC and 29 were healthy controls matched by sex (mostly female), age and ethnicity (mostly Caucasian, only 1 was Hispanic/Latin American and 1 Asian). Of these, 19 had a previous positive history for SARS-CoV-2, but fully recovered from the initial infection, the other 10 had never contracted the SARS-CoV-2 infection, based on negative serology and lack of clinical symptoms compatible with COVID-19 nor positive molecular tests. Clinical characteristics are summarized in Table 1.

**TABLE 1.** Study Population

	Entire Population (N = 90)
Age, median (IQR)	13 (11.75–15.00)
Female, n (%) Male, n (%)	52 (57.8) 38 (42.2)
Ethnicity, n (%) Caucasian Asiatic Sud American	88 (97.8) 1 (1.1) 1 (1.1)
Group Healthy, n (%) Long COVID, n (%)	29 (32.2) 61 (67.8)

Table 2 reports details about the demographic characteristics of children with LC, their symptoms during acute infection, and the most frequent persisting symptoms characteristics of LC. In particular, 80.3% of these presented cardiorespiratory symptoms: [exertional dyspnea (or postexertional malaise), chest pain, tachycardia], 100% presented chronic fatigue, 52.5% presented musculoskeletal symptoms (pain muscle pain at rest and/or under effort, joint pain at rest and/or under effort, muscle fatigue at rest and/or under effort), 47.5% neurological symptoms (headache, difficulty concentrating), 1% gastrointestinal symptoms (abdominal and/or epigastric pain, intestinal changes, recurrent vomiting) and 1.6% dermatological symptoms (rash).

**TABLE 2.** Clinical and Demographics of Long COVID Patients

	LC Patients (N = 61)
Age Mean (±SD) Median (IQR)	13.3 (±2.56) 13 (11.0–14.0)
Female, n (%) Male, n (%)	40 (65.6) 21 (34.4)
Ethnicity, n (%) Caucasian Asiatic Sud American	59 (96.7) 1 (1.6) 1 (1.6)
Acute infection severity, n (%) Asymptomatic Mild Moderate Severe	1 (1.6) 51 (83.6) 9 (14.7) 0 (0.0)
LC Symptoms at follow-up, n (%)	61 (100)
Cardiorespiratory symptoms, n (%)	49 (80.3)
Musculoskeletal symptoms, n (%)	32 (52.5)
Chronic fatigue, n (%)	61 (100)
Neurological symptoms, n (%)	29 (47.5)
Anosmia/dysgeusia, n (%)	3 (4.9)
Gastrointestinal symptoms, n (%)	8 (13.1)
Dermatological symptoms, n (%)	1 (1.6)

### Analysis of the Variables Detected at the Cardiopulmonary Exercise Testing

All LC patients (N = 61) and healthy controls (N = 29) underwent CPET. Children with LC presented a statistically significant higher probability of having abnormal values of peak VO2 (*P* = 0.001), AT% pred (*P* < 0.001), VO2/HR % (*P* = 0.03), VO2 work slope (*P* = 0.002), VE/VCO2 slope (*P* = 0.01). The mean VO2 peak was 30.17 (±6.85) in LC and 34.37 (±6.55) in healthy patients (*P* = 0.007). No statistically significant differences were found in the RER, peak SatO2, predicted breath reserve, and PET CO_2_ values. Details about CPET findings are provided in Table 3.

**TABLE 3.** Analyses of Main Cardiopulmonary Exercise Testing Parameters in Entire Population, Long COVID and Healthy Populations

	Entire Population (N = 90)	Long COVID Population (N = 61)	Healthy Population (N = 29)	*P*
RER, median (IQR)	1.1 (1.05–1.15)	1.12 (1.05–1.16)	1.08 (1.04–1.13)	0.33
RER, n (%) RER maximal RER submaximal	52 (57.8) 38 (42.2)	38 (62.3) 23 (37.7)	14 (48.3) 15 (51.7)	0.21
VO2 peak% pred, median (IQR)	0.74 (0.67–0.85)	0.71 (0.66–0.83)	0.8 (0.72–0.88)	0.07
VO2 peak% pred, n (%) Normal Pathologic	41 (45.6) 49 (54.4)	20 (32.8) 41 (67.2)	21 (72.4) 8 (27.6)	0.001
VO2 peak% pred, n (%)* Mild reduction Moderate reduction Severe reduction	19 (38.8) 24 (49) 6 (12.2)	13 (31.7) 22 (53.3) 6 (14.6)	6 (75.0) 2 (25.0) 0 (0)	0.05
AT% pred, median (IQR)	0.6 (0.39–0.75)	0.56 (0.38–0.72)	0.68 (0.58–0.78)	0.004
AT% pred, n (%) Normal Pathologic	66 (73.3) 24 (26.7)	38 (62.3) 23 (37.7)	28 (96.6) 1 (3.4)	<0.001
VO2/HR% pred, median (IQR)	0.81 (0.74–0.95)	0.82 (0.75–0.95)	0.8 (0.72–0.92)	0.48
VO2/HR% pred, n, (%) Normal Pathologic	62 (68.9) 28 (31.3)	37 (60.7) 24 (39.3)	25 (86.2) 4 (13.8)	0.02
HR, median (IQR)	0.87 (0.82–0.92)	0.87 (0.81–0.90)	0.88 (0.83–0.93)	0.23
HR, n (%) Normal Pathologic	76 (84.4) 14 (16.6)	48 (78.7) 13 (21.3)	28 (96.6) 1 (3.4)	0.03
SatO2 basal, median (IQR)	0.98 (0.97–0.98)	0.98 (0.97–0.98)	0.97 (0.96–0.98)	0.01
SatO2 basal, n (%) Normal Pathologic	90 (100) 0 (0)	61 (100) 0 (0)	29 (100) 0 (0)	–
SatO2 peak, median (IQR)	0.97 (0.96–0.98)	0.97 (0.96–0.98)	0.97 (0.96–0.98)	0.29
SatO2 peak, n (%) Normal Pathologic	89 (98.9) 1 (1.1)	60 (98.4) 1 (1.6)	29 (100) 0 (0)	1
BR% pred, median (IQR)	0.51 (0.42–0.59)	0.55 (0.45–0.63)	0.46 (0.35–0.50)	<0.001
BR% pred, n (%) Normal Pathologic	89 (98.9) 1 (1.1)	60 (98.4) 1 (1.6)	29 (100) 0 (0)	1
VE/VCO2 slope, median (IQR)	29 (25.37–32.25)	30.0 (26.0–34.0)	26.7 (20.05–29.25)	0.005
VE/VCO2 slope, n (%) Normal Pathologic	61 (67.8) 29 (32.2)	32 (52.5) 29 (47.5)	29 (100) 0 (0)	<0.001
VE/VCO2 (AT), median (IQR)	28 (24.92–30.00)	28.0 (26.0–31.50)	25.4 (23.95–29.0)	0.01
PET CO2 (AT), median (IQR)	37 (34.00–41.50)	38.0 (32.50–43.0)	36.0 (35.0–38.0)	0.37

* Performed in N = 49 who presented a pathologic V02 peak pred%.

### Integrated Analysis of the Different Pathophysiologic Patterns of Cardiopulmonary Exercise Testing

Table 4 shows the analysis of the global results of the performed CPET (both in pathological and healthy controls) and the structured analysis for the system responses to physical exercise. Considering “pathological” even apparently normal results for peak VO2 % pred >80% but with findings of anomalies suggestive of muscle deconditioning (VO2/work slope <10 mL/min/watt) especially in symptomatic patients, it emerged that 90.2% of LC patients (55 of 61) had a pathologic test and only 9.8% (6 of 61) had a completely normal test. Instead, in the healthy control group we found 89.7% (26 of 29) of normal tests and only 10.3% of pathological tests (3 of 29). Furthermore, analyzing the results of the CPET based on the VO2 peak% predicted, intended as an expression of the functional capacity of the patients in the 2 groups (LC vs. healthy), statistically significant results emerged (*P* = 0.001), with children with LC having significantly higher pathological values. Statistically significant differences were also highlighted between the 2 groups for oxygen pulse (VO2/HR) indicative of cardiovascular efficiency (*P* < 0.001) and VE/VCO2 slope suggesting of pulmonary vascular involvement (*P* < 0.001) (if VE/VCO2 higher than 30 is suggestive for an underling ventilatory inefficiency or a probably vascular involvement as pulmonary hypertension). Finally, statistically significant differences were also found in regards of muscular component expressed at CPET as VO2/work slope, with significantly fewer children with LC having normal muscular efficiency and more children with LC having muscle deconditioning (*P* = 0.002) and early AT (*P* < 0.001). Nonstatistically significant differences emerged regarding the analysis of the HR and ventilatory component expressed by the respiratory reserve (BR) at CPET.

**TABLE 4.** Cardiopulmonary Exercise Testing Results and Subanalysis of Systemic Dysfunction’s Patterns of in Entire Population, Long COVID and Healthy Populations

	Long COVID Population (N = 61)	Entire Population (N = 90)	Healthy Population (N = 29)	*P*
CPET result, n (%) Normal Pathologic^†^	6 (9.8) 55 (90.2)	32 (35.6) 58 (64.4)	26 (89.7) 3 (10.3)	<0.001
Functional capacity, (VO2 peak% pred) n (%) Normal Mild reduction Moderate reduction Severe reduction	20 (32.8) 14 (23.0) 21 (34.3) 6 (9.8)	41 (45.6) 20 (22.2) 23 (25.6) 6 (6.7)	21 (72.4) 6 (20.7) 2 (6.9) 0 (0)	0.001
Cardiocirculatory efficiency, n (%) Normal Reduced	38 (62.3) 23 (37.7)	67 (74.4) 23 (25.6)	29 (100) 0 (0)	<0.001
Chronotropic competence, n (%) Normal Pathologic	52 (85.2) 9 (14.8)	81 (90.0) 9 (10.0)	29 (100) 0 (0)	0.05
Signs of pulmonary vascular involvement, n (%) No Si	32 (52.5) 29 (47.5)	61 (67.8) 29 (32.2)	29 (100) 0 (0)	<0.001
Likelihood pulmonary vasculopathy*, n (%) Unlikely for IP Suspected of IP Likely for IP	14 (48.3) 14 (48.3) 1 (3.4)	14 (48.3) 14 (48.3) 1 (3.4)	– – –	–
Muscular component, n (%) Muscle efficiency Muscle deconditioning	32 (52.5) 29 (47.5)	57 (63.3) 33 (36.7)	25 (86.2) 4 (13.8)	0.002
Early AT, n (%) No Yes	38 (62.3) 23 (37.7)	67 (74.4) 23 (25.6)	29 (100) 0 (0)	<0.001
Signs of ventilatory limitation, n (%) No Yes	61 (100) 0 (0)	90 (100) 0 (0)	29 (100) 0	–

* Performed in N = 29 of 61 who presented signs of pulmonary vascular involvement (VE/VCO2 slope >30) according matching between VE/VCO2(AT) and PETCO2(AT).

† For pathological CPET here we considered also pz with normal VO2 peak but with very low VO2 work slope and/or an early AT suggestive of a muscle deconditioning condition.

### Analysis of Phenotypes in Long COVID Patients According to Cardiopulmonary Exercise Testing Results

**FIGURE 1.**
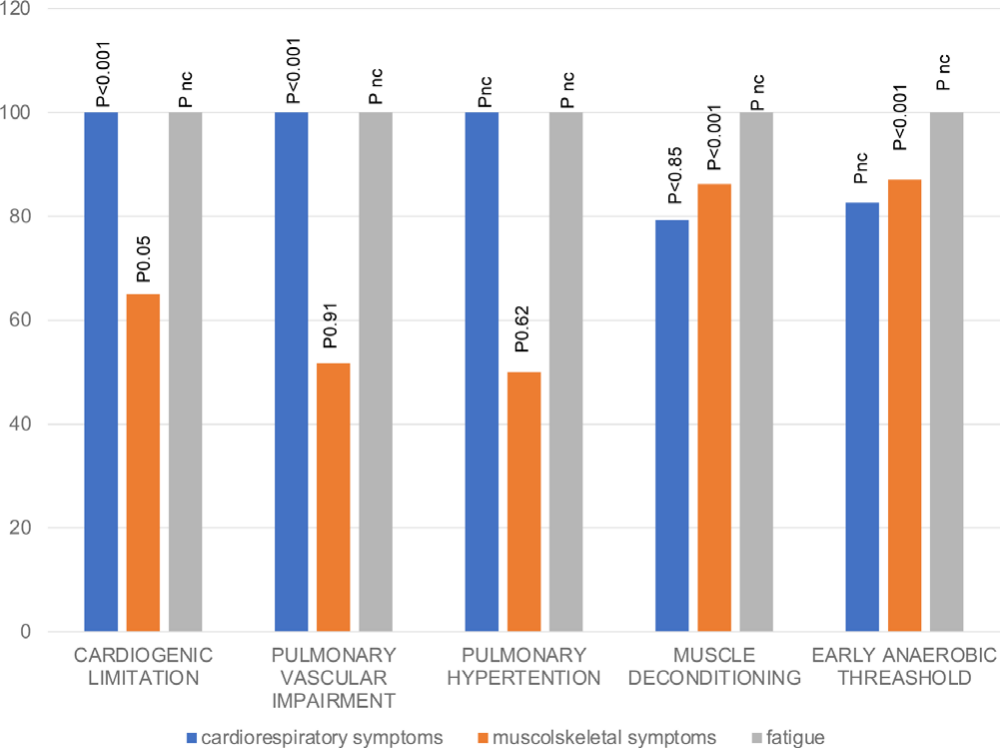
CPET findings according to main cluster of reported symptoms in patients with Long COVID.

We attempted to correlate “clinical phenotypes” in children with LC with findings at CPET. In particulars, we correlated the presence of cardiorespiratory symptoms (dyspnea, tachycardia and chest pain), musculoskeletal (joint and muscle pain at rest and under stress) and asthenia, to CPET findings of cardiovascular response, signs of pulmonary vascular involvement with underlying probability of pulmonary hypertension based on the interpolation of data between VE/VCO2 (AT) and PETCO2 (AT), muscle deconditioning and early AT to find an objective pathophysiological correlation (Fig. 1).

We found that the presence of cardiogenic limitation (expressed by the analysis of VO2/HR and HR), or signs of pulmonary vascular involvement (expressed by VE/VCO2 slope intercept values >30 in the absence of signs of ventilatory inefficiency) on CPET were significantly associated with the presence of persistent cardiorespiratory symptoms and asthenia (*P* < 0.001). Conversely, the presence of muscle deconditioning and early AT on CPET were significantly associated with the presence of persistent musculoskeletal symptoms (*P* < 0.001).

Signs of pulmonary hypertension on CPET [based on the interpolation of the VE/VCO2 (AT) and PETCO2 (AT) values] were more frequently seen in children with cardiorespiratory symptoms and asthenia, although associations were not statistically significant.

## DISCUSSION

To our knowledge, this is the largest case-controlled study documenting that children with LC have objective pathological findings at CPET compared with controls. Children with LC have a reduced VO2 peak (Oxygen uptake at peak of exercise), abnormal cardiovascular efficiency (VO2/HR% pred), pathological VE/VCO slope (indicative of the possible presence of ventilatory inefficiency/pulmonary vascular commitment if higher than 30), and abnormally reduced slope of VO2 work (indicative of muscle deconditioning). In addition, we stratified the probability that an altered VE/VCO2 slope was indicative of an underlying pulmonary vasculopathy such as pulmonary hypertension by combining the parameters of VE/VCO2 (AT) and PETCO2 (AT) measured at anaerobic threshold value, and it emerged that 48% of the LC patients had a suspicious phenotype for pulmonary hypertension. Conversely, heart rate analyses and parameters of respiratory function [expressed by a normal breathing reserve (BR) and absence of desaturation of SpO2 at peak of exercise] were similarly normal in cases and controls.

Altogether, our study opens a new scenario in terms of diagnostic possibilities for children with suspected LC, but also a new hypothesis in terms of mechanisms leading to common symptoms in LC, like fatigue and exercise intolerance, suggesting that pediatric LC is a real disease and not a psychologic consequence of the pandemic.^19^ In fact, our findings are in line with studies investigating the role of CPET in adults with LC.^20–25^ These data have implications as they document that symptoms are real and not psychologic, may allow further diagnostics within research contexts, allow doctors to produce certificates to support patients for adapted activities (eg, school attendance or sport for children) or for insurance policies in countries with insurance-based healthcare systems.

In adults with LC and absence of organic damage when investigated with first-line diagnostics,^26^ CPET represents a useful standardized tool for measuring exercise capacity and aiding in the differential diagnosis of exercise limitations.^17,27,28^ Case series suggested that SARS-CoV-2 infection is associated with reduced exercise capacity.^29,30^ Two reviews and one meta-analysis found that adults with LC have reduced exercise capacity at CPET compared with recovered individuals.^31–33^ Muscular and/or peripheral oxygen extraction abnormalities were also commonly reported. However, distinguishing deconditioning from altered oxygen delivery, mitochondrial dysfunction and muscular pathology, can be challenging with noninvasive CPET (invasive CPET is difficult to be done in children) without adjunctive testing or without having pre-COVID-19 CPET for comparison. Anyway, all these mechanisms have been studied as possibly linked with symptoms of LC.^34^

In addition to muscular decondition, our study found that a subgroup of patients has CPET documentation of cardiovascular limitation leading to exercise intolerance (many children, in fact, were not able to conclude the test). These events during LC are more probably due to poorly characterized functional events, like autonomic dysfunction or other unknown factors, rather than macroscopic heart damage. Studies in adults, in fact, found that cardiac autonomic dysregulation is a major cause of exercise intolerance, leading to findings of cardiovascular limitation at CPET.^35^ In another study,^36^ we documented significant differences in heart rate variability of children with LC compared with controls. Modulation of the HR during exercise is a dynamic process tightly regulated by the autonomic nervous system and its imbalance may manifest during exercise as chronotropic incompetence or inadequate HR recovery. Some CPET studies reported chronotropic incompetence,^35,37^ while others observed an abnormal HR recovery.^38–40^ In our study HR was normal, while chronotropic incompetence was sometimes found. Autonomic dysfunction and endothelial dysfunction are possible mechanisms for these findings and could be caused by SARS-CoV-2 infection of neurons and endothelial cells, chronic inflammation or autoimmune mechanisms. Dysfunctional breathing may also be a manifestation of dysautonomia. The autonomic nervous system and endothelial interaction may regulate peripheral vasomotor tone; together, they may explain differences in peripheral extraction and preload failure. Small-fiber neuropathy among individuals who have LC symptoms with postural orthostatic tachycardia syndrome may be associated with reduced cerebral blood flow and postural symptoms. All together, these events may possibly be the cause of the abnormal cardiovascular efficacy we documented at the CPET of children with LC. These data would suggest that the recognition of specific subphenotypes at CPET (eg, suggestive of dysautonomia) can suggest the need to assess heart rate variability, as we have documented in a previous case report and in a larger original study^13,36^ and recognize syndromes associated with inappropriate tachycardia, which are potentially treatable, also in children.^41^ Obviously, more studies are needed to validate our observations.

In our cohort, some children had CPET findings indicative of pulmonary hypertension. As well documented by adult studies, CPET is a well-established noninvasively tool that provides information about body’s exercise capacity related to the pathophysiology of pulmonary arterial hypertension.^18,42^ In fact, in the presence of typical findings in results of CPET (such as reduced VO2 peak, an high VE/VCO2 slope and low PET CO_2_ at anaerobic threshold) we can reasonably differentiate a pulmonary vascular limitation to exercise and aid diagnosis in patients with exertional dyspnea, especially in patients with a high-risk population, providing an earlier detection in patients more likely to have pulmonary hypertension before resting adaptation become apparent (this could explain the normal results seen in our echocardiograms).

Direct ventilatory, pulmonary vascular, and cardiac limitations were uncommon CPET findings in our patients with LC as well as other adult studies,^31–33^ suggesting that direct heart or lung damage are not major drivers of exercise limitations in LC, as also documented by the fact that most patients have normal baseline investigations like hearth ultrasound or chest CT scan (also our patients performed hearth ultrasound, which was normal in all evaluated patients).

Our study also aimed to subclassify LC patients according to the main symptoms (cardiorespiratory, musculoskeletal and asthenia) and the CPET results. The results showed that LC patients who presented mostly cardiorespiratory symptoms had a corresponding pattern of functional limitation at CPET characterized by signs of cardiogenic inefficiency and possible pulmonary vascular involvement; on the other hand, those who mostly presented asthenia (100%) and musculoskeletal symptoms (86%) presented a corresponding CPET pattern suggestive of signs of muscle deconditioning as well as an early anaerobic threshold. These data are in line with recent hypotheses suggesting that LC and myalgic encephalomyelitis/chronic fatigue syndrome are syndromes presenting with different clinical phenotypes that may deserve different therapeutic approaches or inclusion criteria for pharmacologic trials.^43,44^

We have not found other large pediatric studies evaluating CPET in children with LC to compare our findings. We only found a pediatric study investigating CPET in multisystem inflammatory syndrome survivors.^45^ CPET showed lower-than-expected peakVO2 and peak-oxygen-pulse values (50% of cases) and higher-than-expected VE/VCO2-slope values (95% of cases).

Our study has limitations to address. Although this is the largest case-controlled study investigating CPET in children with LC, the cohorts are relatively small. This is particularly true for the control group, and was mostly due to the policies that limited the number of tests we could do with a machine requiring deep breathing (and therefore high risk of nosocomial transmission of SARS-CoV-2) during most of the pandemic periods. In addition, we could not include children younger than 10 years or shorter than 140 cm, as our machine was set for older children and adults. We do not have transcriptomic/metabolic/bioptic correlates of the findings we found on CPET. Last, many studies have demonstrated that a 2-day CPET study can be even more useful to document postexertional malaise in LC and myalgic encephalomyelitis/chronic fatigue syndrome patients, however, our resources did not allow that. Importantly, adding another control group of children with non-COVID postinfectious fatigue and/or exercise intolerance would have been helpful to know whether this entity specific to COVID-19. We have already planned similar studies, and we hope that all centers caring for children and adolescents with chronic fatigue syndrome will establish similar research protocols.

In conclusion, our study, based on a rigorous definition of LC through in-patient assessment, showed that children with LC have objective impaired functional capacity (expressed by a low VO2 peak) when assessed with CPET compared with controls. Children with LC are characterized by the presence of signs of muscle deconditioning and, less frequently, signs of cardiogenic inefficiency (represented by low value of pulse of oxygen), as the most impactful contributions to exercise intolerance. This test is not only useful for diagnostic and insurance reasons, but may also be useful to better characterize patients for inclusion in clinical trials and to monitor therapeutics with objective outcome measures.
